# Discovering Associations in Biomedical Datasets by Link-based Associative Classifier (LAC)

**DOI:** 10.1371/journal.pone.0051018

**Published:** 2012-12-05

**Authors:** Pulan Yu, David J. Wild

**Affiliations:** School of Informatics and Computing, Indiana University, Bloomington, Indiana, United States of America; University of Glasgow, United Kingdom

## Abstract

Associative classification mining (ACM) can be used to provide predictive models with high accuracy as well as interpretability. However, traditional ACM ignores the difference of significances among the features used for mining. Although weighted associative classification mining (WACM) addresses this issue by assigning different weights to features, most implementations can only be utilized when pre-assigned weights are available. In this paper, we propose a link-based approach to automatically derive weight information from a dataset using link-based models which treat the dataset as a bipartite model. By combining this link-based feature weighting method with a traditional ACM method–classification based on associations (CBA), a Link-based Associative Classifier (LAC) is developed. We then demonstrate the application of LAC to biomedical datasets for association discovery between chemical compounds and bioactivities or diseases. The results indicate that the novel link-based weighting method is comparable to support vector machine (SVM) and RELIEF method, and is capable of capturing significant features. Additionally, LAC is shown to produce models with high accuracies and discover interesting associations which may otherwise remain unrevealed by traditional ACM.

## Introduction

Chemical and biological data contain information about various characteristics of compounds, genes, proteins, pathways and diseases. Thus a wide spectrum of data mining methods is used to identify relationships in these large and multidimensional datasets and to generate predictive models with high accuracy and interpretability. Recently, *associative classification mining (ACM)* has been widely used for this purpose [1]–[4]. ACM is a data mining framework utilizing association rule mining (ARM) technique to construct classification systems, also known as associative classifiers. An associative classifier consists of a set of classification association rules (CARs) [5] which have the form of X→Y whose right-hand-side Y is restricted to the classification class attribute. X→Y can be simply interpreted as if X then Y. ARM is introduced by Agrawal et al [6] to discover CARs which satisfy the user specified constraints denoted respectively by minimum support (*minsup*) and minimum confidence (*minconf*) threshold. Given a dataset with each row representing a compound, each column (called as item, feature or *attribute*) is a test result of this compound on a tumor cell line and all compounds are labeled as active or inactive *class*, a possible classification association rule can be {MCF7 inactive, HL60 (TB) inactive → inactive} with support = 0.6 and confidence = 0.8. This particular rule states that when a compound is inactive to both MCF7 cell line and HL60 (TB) cell line, it tends to be inactive. The support, which is the probability of a compound being inactive to both MCF7 and HL60 (TB) and being classified as inactive together, is 0.6; the confidence, which is the probability of a compound to be inactive given inactive to both MCF7 and HL60 (TB), is 0.8. In ACM, the relationship between attributes and class is based on the analysis of their co-occurrences within the database so it can reveal interesting correlations or associations among them. For this reason, it has been applied to the biomedical domain especially to address gene expression relations [7]–[11], protein-protein interactions [12], protein-DNA interactions [13], and genotype and phenotype mapping [14] *inter alia*.

Traditional ACM does not consider feature weight, and therefore all features are treated identically, namely, with equal weight. However, in reality, the importance of feature/item is different. For instance, {beef → beer} with support = 0.01 and confidence = 0.8 may be more important than {chips → beer} with support = 0.03 and confidence = 0.85 even though the former holds a lower support and confidence. Items/features in the first rule have more profit per unit sale so they are more valuable. Wang et al [15]–[17] proposed a framework called weighted association rule mining (WARM) to address the importance of individual attributes. The main idea is that a numerical attribute can be assigned to every attribute to represent its significance. For example, {Hypertension = yes, age>50→ Heart_Disease} with {Hypertension = yes, 0.8}, {age>50, 0.3} is a rule mined by WARM. The importance of hypertension and age >50 to heart disease is different and denoted by value 0.8 and 0.3 respectively. The major difference between ARM and WARM is how the support is computed. Several frameworks are developed to incorporate weight information for support calculation [15]–[22]. Studies have been carried out on WARM by using pre-assigned weights. Nonetheless, most datasets do not contain those pre-assigned weight information.

**Figure 1:**
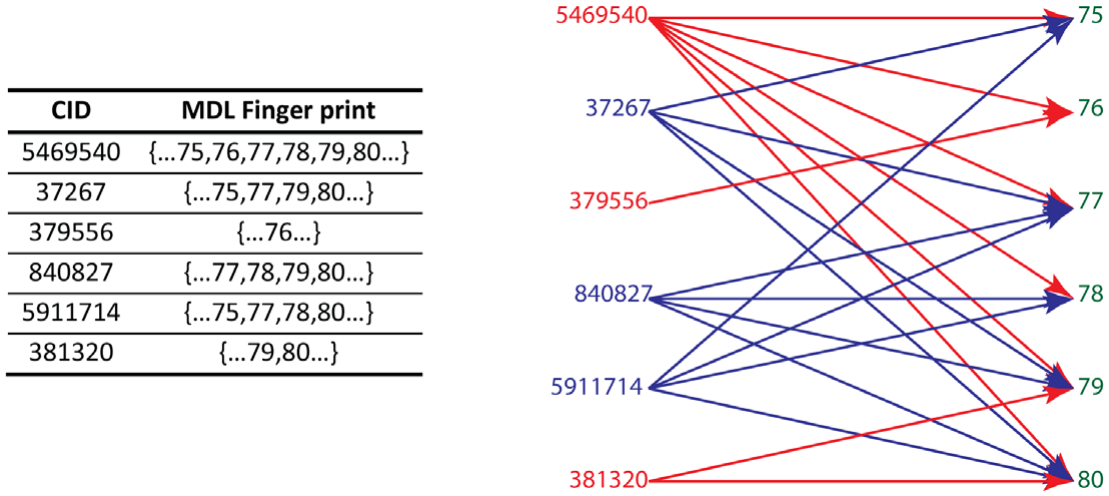
The bipartite model of a dataset. (The bipartite model is also a heterogeneous system. Blue represents active compounds and red for inactive compounds with both contributing to the green node-feature/attribute.).

In machine learning, feature selection and feature weighting are broadly used to deal with the significance of features and derive weight information automatically from a dataset itself. Feature selection is a technique of selecting a subset of relevant features by removing low significant features; feature weighting is a technique of approximating the optimal degree of influence of individual features. Feature weighting preserves all features by assigning smaller weight to relatively insignificant features and has the advantage of taking into account of all features as well as not requiring searching an appropriate cut-threshold [23]. In some circumstances, it might be the only option when eliminating features with a low contribution to classification is inappropriate. Especially, to understand the overall relationship between genes and a disease, a small subset of genes although having good prediction ability may not have sufficient discriminating power [24]. Like feature selection, feature weighting approaches fall into two categories: 1) filter methods which are performed in a pre-processing step before modeling; 2) wrapper methods which are iterative and generally use the same learning algorithm as modeling. In wrapper methods, the evaluation result of relevancy is used for feature weighting. Usually, wrapper methods perform better than filter methods while filter methods are faster and cheaper.

Sun et al. [25] proposed a link-based filter feature weighting approach. The weights are derived from the dataset itself by extending Kleinberg’s HITS (Hyper Induced Topic Selection) model [26] and algorithm on bipartite graphs. HITS and PageRank are two major link-based ranking algorithms. PageRank is developed by Brin and Page [27] and has been commercially successfully used in the search engine Google. HITS ranks webpages by analyzing the in-links and out-links. Webpages pointed to by many other pages are defined as “authority” while webpages linked to many other pages are called “hub”. HITS emphasizes the notion of “mutual reinforcement” between the “authority” and “hub”. Its intuitive interpretation is that a good “authority” is pointed to by a lot of good “hubs” and a good “hub” points to many good “authorities”. PageRank uses a very similar idea that a “good” webpage should be linked or link to other “good” webpages. Unlike the “mutual reinforcement” approach, it focuses on hyperlink weight normalization and web surfing based on random walk models. Both approaches have pros and cons. The computation of PageRank is stable and its behavior is well-defined due to the probabilistic interpretation. Furthermore, PageRank can be used on large page collections because even though the larger communities will affect the final ranking, they will not overwhelm the small ones. In contrast, HITS is not stable and cannot be applied to large page collections since only the largest web community will influence the final ranking. However, it can capture the relationships among the webpages with more details [28]. Hence, an algorithm capable of integrating both HITS and PageRank may improve Sun’s weighting method.

The general PageRank cannot be applied to bipartite graphs as it produces different rankings for webpages with the same in-links [29], as a result, a better ranking scheme is needed for ranking in bipartite graphs while integrating PageRank and HITS [30]. The SALAS (stochastic approach for link structure analysis) [31]–[33] combines the random surf model of PageRank with hub/authority principle of HITS. It generates a bipartite undirected graph **H** based on the web graph **G**. One subset of **H** contains all the nodes with positive in-degree (the potential “authorities”) and the other subset consists of all the nodes with positive out-degree (the potential “hubs”). A travel is completed by a two-step random walk. For example, from the “hub” to the “authority” and from the “authority” back to the “hub”. As in the PageRank, each individual walk is a Markov process with a well-defined transition probability matrix [31]. Nevertheless, besides SALAS does not really implement the “mutual reinforcement” of HITS because the scores of both authority and hub are not related by the hub to authority and authority to hub reinforcement operations, its score propagation differs from HITS (a similarity-mediated score propagation). Moreover, its random walk model does not directly simulate the behavior of the surfer in PageRank either. For SALAS, a surfer can jump from webpage *p_i_* to *p_j_* even though there is no hyperlink between them, and there is no link-interrupt jumps. Based on a similar approach as SALAS, Ding et al proposed a unified framework integrating HITS and PageRank [34].

**Figure 1** indicates that a database can be represented by a bipartite graph equally [25]. In the graph, left is the table layout representation and can be represented by the bipartite graph on the right. Compounds and features linked to each other can be viewed as webpages. As a consequence, the link-based algorithms used to rank the webpage such as HITS or PageRank can be utilized to rank compounds or features. The algorithms say that if a webpage has many important links to it, the links from it to other webpages become important too. For our case, this means a highly weighted compound should contain many highly weighted features and a highly weighted feature should exist in many highly weighted compounds. Accordingly, the ranking score can be used for feature weighting. Although Ding’s unified framework can be used to derive the ranking score automatically, it cannot distinguish the contributions of different types of connections. For chemical dataset mining, each chemical feature may connect to both active and inactive compounds; for biological dataset mining, each gene may connect to a disease either as suppressor or activator. Chemical features existing frequently in active compounds or genes major associated with suppressors are more interested in. In **Figure 1**, when we consider the contribution of compounds to the weight of a node/attribute 78, we want to distinguish the contribution of compound 5469540 from the contribution of compound 840827 and 5911714. Ding’s unified framework treats the contribution of the nodes equally as a homogenous system [34]; Chen et al developed a framework calculating the weight for either homogenous or heterogeneous systems [35]. In Chen’s model, connections can have different impacts on a node.

In this paper, we describe a link-based unified weighting framework which combines the mutual reinforcement of HITS with hyperlink weighting normalization of PageRank based on Ding and Chen’s frameworks, resulting in highly efficient link-based weighted associative classifier mining from biomedical datasets without pre-assigned weight information.

Our main contributions are: 1) development of a novel link-based weighting scheme for mining biomedical datasets; 2) implementation of a novel link-based associative classifier by combining the feature weighting method, weighted association rule mining (WARM) and the CBA algorithm [5]; 3) application of this method to two important biomedical datasets.

In the following sections, the dataset, link-based feature weighting, WARM and algorithm of LAC will be discussed, followed by the application of LAC to two datasets. In the end, we present our conclusions and future work.

**Table 1:** A compound dataset encoded by MDL public keys.

CID	MDL Finger print
C1	{…81,82,83,84…}
C2	{…82,84…}
C3	{…81,84…}
C4	{…81,82,84,85…}
C5	{…81,82,83,84,85…}
C6	{…82,83,85…}

**Table 2:** MDL public keys and their weight.

Feature	Weight
81	0.8
82	1
83	0.8
84	1.6
85	1

**Table 3:** Supports and types of itemsets (frequent or not).

Itemset	Classical		Weighted		Adjusted Weighted	
	Support	Frequent	Support	Frequent	Support	Frequent
81	0.67	Y	0.53	Y	0.75	Y
83	0.50	Y	0.4	Y	0.66	Y
81 83	0.33	Y	**0.27**	**N**	**0.44**	**Y**
83 84	0.33	Y	**0.27**	**N**	**0.44**	**Y**
81 84	0.67	Y	0.8	Y	0.75	Y
81 83 84	0.33	Y	0.35	Y	0.44	Y

## Materials and Methods

### 1. Data Set

LAC is applied to two datasets: a. Ames mutagenicity dataset [36], b. NCI-60 tumor cell line dataset [37]. In Ames dataset, there are 6,512 compounds provided in SMILES format and is benchmarked by SVM, Random Forests, k-Nearest Neighbors, and Gaussian Processes. The authors used 5-fold cross validation to evaluate the generated models. The area under this ROC-Curve (AUC) is utilized to assess the performance which ranges from 0.79 to 0.86. The GI50 data of NCI-60, which is the concentration of the anti-cancer drug that inhibits the growth of cancer cells by 50%, is used and processed as following. First, among the 60 tumor cell lines, IGR-OV1, MDA-MB-468 and MDA-N are removed due to too many missing values. Then, compounds having missing values are also discarded. In the final dataset, 5,937 compounds with 57 bioassay results in total are included. For the Ames dataset, if a compound is positive, it is carcinogenic; for the NCI-60, the compound is “active” only if its GI 50 is greater than 5.

**Figure 2:**
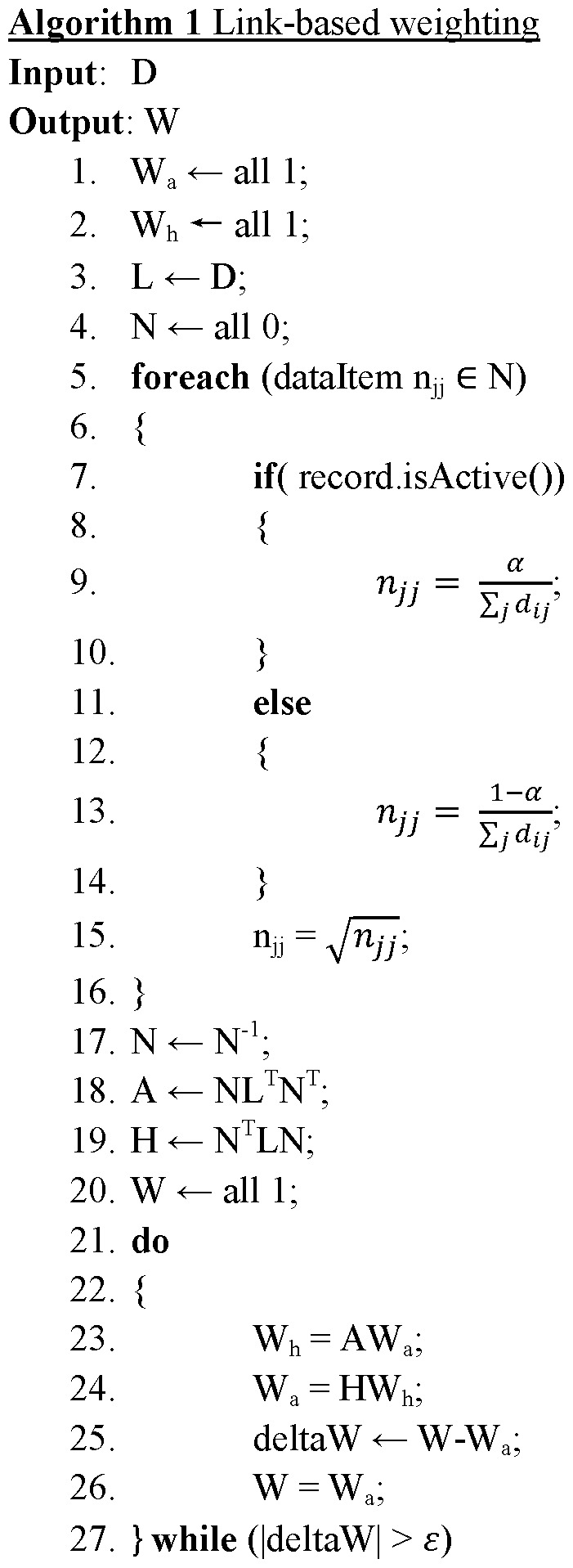
Link-based weighting.

**Figure 3:**
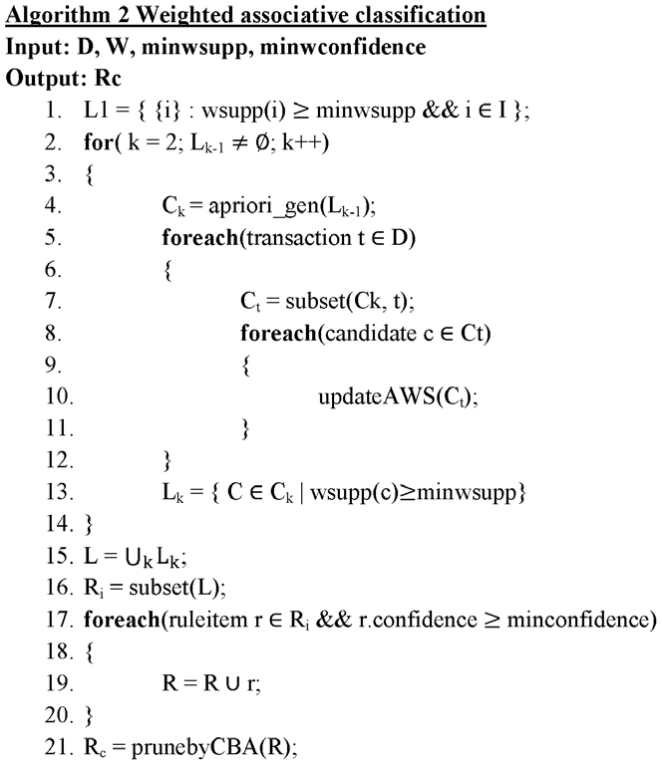
Weighted associative classification.

### 2. MDL Public Keys

MDL public key set also called MACCS key set is a 166-bit string with each bit encoding a predefined chemical structure feature. MDL public keys are extensively used in biomedical research due to their relatively high performance and the one-to-one map between the structural feature and fingerprint [37], [38]. The fingerprint is computed by using the CDK [39] software package and reformatted for LAC.

**Figure 4:**
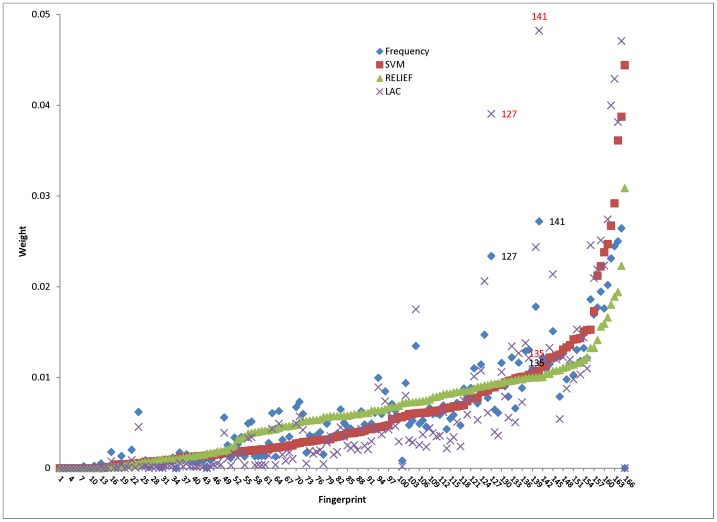
Results of different weighting methods.

**Table 4:** Correlation analyses of the weighting results.

		Frequency	SVM	RELIEF	LAC
**Frequency**	Pearson Correlation	1	.776**	.791**	.947**
	Sig. (2-tailed)		.000	.000	.000
**SVM**	Pearson Correlation	.776**	1	.949**	.759**
	Sig. (2-tailed)	.000		.000	.000
**RELIEF**	Pearson Correlation	.791**	.949**	1	.712**
	Sig. (2-tailed)	.000	.000		.000
**LAC**	Pearson Correlation	.947**	.759**	.712**	1
	Sig. (2-tailed)	.000	.000	.000	

** Correlation is significant at the 0.01 level (2-tailed).

### 3. Bio Fingerprint

Bioassay readouts have been used as features (“biospectra” or “bio fingerprint”) for data mining in several studies and produced high quality models [40], [41]. These bioactivity profiles link the potential targets with the chemical compounds and provide insights into the relationships among diseases, compounds and bioactivities. In this study, results of related bioassay analyses are used as features for the classification of chemical compounds. Each GI50 value is transformed into “active” (GI50 is greater or equal than 5) or “inactive” (GI50 is less than 5). The T-47D is used as a label class and the results from other cell lines are used as features.

**Table 5:** The rankings of chemical features from frequency and LAC.

Bit	Frequency	LAC	Bit	Frequency	LAC	Bit	Frequency	LAC	Bit	Frequency	LAC
1	1	1	43*	24	19	**85**	**69**	**78**	126	110	101
2	1	1	**44**	**4**	**6**	**86**	**68**	**71**	127	152	152
**3**	**3**	**5**	**45**	**27**	**30**	**87**	**63**	**66**	128*	100	80
4	1	1	**46**	**33**	**41**	**88**	**65**	**68**	129*	94	77
5	1	1	47	44	44	89*	96	93	**130**	**129**	**130**
6	1	1	48*	40	39	90*	73	67	131*	118	111
7	1	1	**49**	**85**	**109**	91*	66	61	132*	111	91
8	12	12	50*	51	48	**92**	**77**	**83**	**133**	**134**	**141**
9	1	1	51*	32	26	**93**	**93**	**96**	134*	102	98
10	1	1	**52**	**56**	**75**	**94**	**121**	**131**	**135**	**130**	**140**
11	13	13	53*	52	50	95	88	88	136*	117	112
12	1	1	**54**	**58**	**62**	**96**	**114**	**117**	137	137	137
**13**	**16**	**18**	55*	35	31	97	99	99	138*	139	129
14	8	8	**56**	**76**	**108**	**98**	**106**	**107**	139*	123	115
15*	5	3	**57**	**79**	**89**	99*	98	94	**140**	**147**	**148**
**16**	**47**	**53**	58*	37	35	100	82	82	141	156	156
17	7	7	**59**	**36**	**38**	**101**	**23**	**25**	**142**	**133**	**135**
18	2	2	60*	39	34	**102**	**119**	**127**	143*	124	122
**19**	**38**	**43**	61*	41	36	**103**	**72**	**79**	**144**	**128**	**134**
20*	6	4	**62**	**53**	**55**	104*	80	70	**145**	**143**	**145**
**21**	**15**	**16**	**63**	**92**	**114**	**105**	**141**	**143**	146*	135	128
**22**	**48**	**54**	64*	34	33	106*	75	73	147*	112	92
23	14	14	**65**	**97**	**106**	107	81	81	**148**	**136**	**138**
**24**	**95**	**113**	66*	54	49	108*	70	58	149*	120	110
**25**	**20**	**28**	67*	49	46	109*	103	87	150*	126	123
**26**	**25**	**27**	**68**	**59**	**69**	**110**	**89**	**90**	**151**	**122**	**124**
27*	10	9	**69**	**50**	**51**	111*	91	84	152*	138	132
**28**	**18**	**23**	**70**	**104**	**118**	112*	87	72	153*	131	120
29*	19	15	**71**	**109**	**121**	113*	105	104	154*	140	126
30	11	11	**72**	**90**	**95**	114*	67	60	155*	132	119
**31**	**9**	**10**	73*	45	40	115*	83	64	**156**	**148**	**149**
32	29	29	74*	60	52	116*	86	74	157*	144	139
**33**	**30**	**32**	**75**	**57**	**65**	117*	108	103	158	146	146
34	31	20	76*	61	57	118*	71	63	159*	149	144
35	1	1	**77**	**64**	**76**	**119**	**115**	**125**	160*	145	142
**36**	**46**	**47**	78	42	42	120*	113	105	161	150	150
37	26	24	**79**	**74**	**86**	121	116	116	**162**	**151**	**153**
**38**	**43**	**45**	**80**	**84**	**100**	**122**	**125**	**133**	**163**	**153**	**154**
39	21	21	**81**	**55**	**56**	123*	107	85	164*	154	151
40	22	22	82*	62	59	**124**	**127**	**136**	165	155	155
41	17	17	**83**	**101**	**102**	**125**	**142**	**147**	166	1	1
**42**	**28**	**37**	**84**	**78**	**97**						

* means the ranking in the frequency is higher than that in LAC otherwise bold, and the rest means the same.

For each of the 6,512 compounds in Ames data, we attempt to predict whether it is carcinogenic or not based on the MDL public keys. For the 5,937 compounds in NCI 60, we first use Bio fingerprint to predict whether they are agonist or antagonist to T-47D cell line. Then, for those 3,199 compounds in the NCI-60 dataset having 2D structures available in the downloaded structure file, a hybrid fingerprint is generated by combing MDL public keys and Bio fingerprint to build models.

**Table 6:** The modeling results.

Model#	RELIEF	SVM	Frequency	CBA	LAC	Bio fingerprint	MDL_Bio fingerprint
1	89.71%	89.71%	91.70%	93.39%	92.93%	100.00%	99.69%
2	89.09%	89.40%	90.63%	91.40%	91.40%	100.00%	100.00%
3	88.63%	88.63%	89.71%	90.32%	91.71%	99.33%	100.00%
4	87.86%	88.79%	88.79%	88.17%	91.71%	100.00%	100.00%
5	90.02%	90.02%	90.17%	90.48%	90.78%	100.00%	99.06%
6	86.64%	86.94%	88.02%	88.48%	90.32%	100.00%	100.00%
7	91.09%	91.40%	91.86%	90.63%	92.78%	100.00%	99.69%
8	88.63%	88.79%	88.79%	89.55%	90.63%	100.00%	100.00%
9	89.25%	89.40%	90.48%	91.86%	91.55%	100.00%	100.00%
10	89.55%	89.55%	90.94%	92.01%	91.86%	100.00%	99.06%
**Average**	**89.05%**	**89.26%**	**90.11%**	**90.63%**	**91.57%**	**99.93%**	**99.75%**

Let **L** =  (L_ij_) be the adjacency matrix of the web graph **G** =  (V,E), where V is the set of webpages and E is the set of links between them. L_ij_ = 1 if page *i* links to page *j* and L_ij_ = 0 otherwise. L^T^ will be the transpose of L. If the graph is directed, the in-degree matrix D_in_ and out-degree matrix D_out_ are also defined. Given vectors **d_in_** = (b_1_, b_2_, …, b_n_)^T^ where b_j_ is the in-degrees of page j(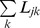) and **d_out_** = (o_1_,o_2_, …, o_n_)^T^ where o_j_ is the out-degrees of page j 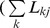). D_in_ is a diagonal matrix denoted as D_in_ = diag(**d_in_**) and D_out_ = diag(d**_out_**).

**Table 7:** Top 20 rules from frequency and LAC classifier.

Number	Frequency	LAC
1	157,140,93 ->positive	**155,140,62 ->positive**
2	139,124,104 ->positive	**140,62 ->positive**
3	157,155,93 ->positive	**132,69 ->positive**
4	157,93 ->positive	**140,118,69 ->positive**
5	157,140,123 ->positive*	**155,62 ->positive**
6	163,140,93 ->positive*	**157,140,69 ->positive**
7	118 ->positive	**157,62 ->positive**
8	155,140,93 ->positive	**158,140,69 ->positive**
9	157,155,123 ->positive*	**62 ->positive**
10	157,123 ->positive	**155,118,69 ->positive**
11	144,124,104 ->positive	**158,157,69 ->positive**
12	155,140,123 ->positive*	**157,118,69 ->positive**
13	157,155,124 ->positive*	**140,69 ->positive**
14	140,101 ->positive	**132,121,70 ->positive**
15	161,139,104 ->positive	**157,132,70 ->positive**
16	157,126,124 ->positive*	**132,70 ->positive**
17	124,104 ->positive*	**140,129,70 ->positive**
18	139,126,124 ->positive*	**157,129,70 ->positive**
19	129,123 ->positive*	**161,157,23 ->positive**
20	144,139,124 ->positive	**157,126,23 ->positive**

* is exclusively in the frequency approach, bold only in LAC and others are common ones.

### 4. HITS

In HITS, vectors **x** = (x_1_,x_2_,…,x_n_)^T^ and **y** = (y_1_,y_2_,…y_m_)^T^ represent the scores of authority and hub respectively. HITS defines recursive equations as following:
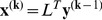
(1)

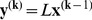
(2)Where k
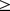
1 and y^(0)^ = **e**, e is a vector of all 1s and **x^(k)^** denotes k-th iteration. **Equation 1** tells that authoritative pages are those linked by good hub pages, and **equation 2** means good hubs are pages that link to authoritative pages. It can be rewritten as:



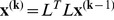
(3)

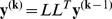


**Table 8:** Selected Top 5 active rules using bio fingerprint.

Number	Rules	Support	Confidence
1	MCF7 inactive, HL60(TB) inactive → inactive	29.1%	95.8%
2	MCF7 inactive, MOLT-4 inactive →inactive	29.7%	95.8%
3	MCF7 inactive,CCRF inactive →inactive	28.7%	95.4%
4	MCF7 inactive, K-562 inactive →inactive	30.7%	95.4%
5	MCF7 inactive, RPMI-8226 inactive →inactive	31.9%	95.2%
…	…	…	…

### 5. PageRank

In PageRank, given **x** = (x_1_,x_2_,…,x_n_)^T^, x_i_ is the PageRank of page *i*; the recursive PageRank equation is defined in matrix notation as:
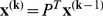
(5)where P =  (P_ij_) is a **stochastic matrix** (the sum of every column equals to 1) with P_ij_ = 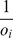. P^T^ can be expressed as:



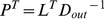
(6)If considering the link-tracking jump and link-interrupt jump, the full transition probability can be written as:
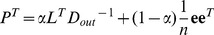
(7)where 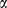 is the damp factor from 0 to 1.

As the way processed in SALAS, if the web graphs are transformed into bipartite graphs, the above x will be the authority score and the hub score y can be defined as:
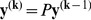
(8)

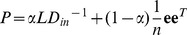
(9)


**Figure 5:**
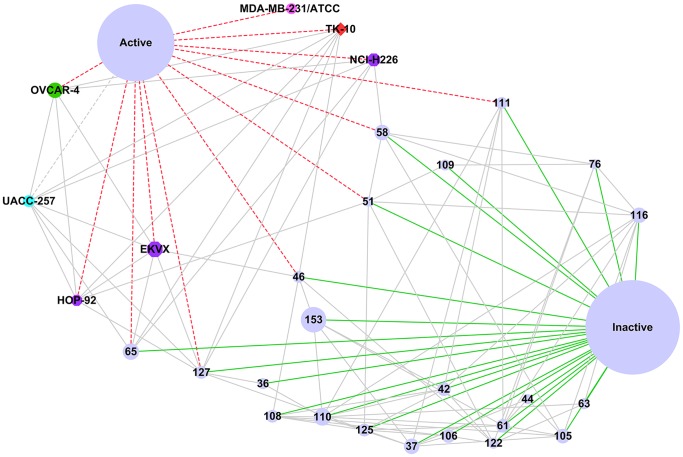
The connections between chemical features and cell lines. (Red dot means a connection to active; green solid to inactive; light gray means features associated to each other. Purple: Non-small cell lung; Red: Renal; Pink: Breast cancer; Green; Ovarian and Light blue; Melanoma.).

Comparing the equations between HITS and PageRank (**equation 1** & **2** versus **5** & **8**), it is possible that a unified framework can be derived to combine advantages from both HITS and PageRank.

**Table 9:** Top 5 rules using the combined fingerprint.

Number	Rules	Support	Confidence
1	MCF7 active, bit 29 → active	2.0%	98.2%
2	SK-MEL-2 active, bit 29 →active	1.8%	98.11%
3	UACC-62 active, bit 33 → active	2.0%	97.7%
4	NCI-H226 active, bit 33 → active	1.7%	97.3%
5	HCC-2998 active, bit 33 → active	1.6%	97.2%

### 6. Unified Framework

If we define the L^T^L in **equation 3** and P^T^ in **equation 5** as operation A^op^ (authority) and LL^T^ in **equation 4** and P in **equation 8** as operation H^op^ (hub). The critical component of the framework is to define the new A^op^ and H^op^. Ding’s implementations of A^op^ and H^op^ [34] are used here since it generalizes the features of HITS and PageRank and combines them together.

Chen’s model [35] divided the web pages into homogenous and heterogeneous systems so the scores of authority and hub contain the reinforcement of links from both systems. Different weights can be assigned to homogenous or heterogeneous systems to adjust the importance of their links in the final ranking. Similarly, in our case, the nodes, such as compounds, are classified as active/inactive or positive/negative thus the dataset is converted to a heterogeneous system. The relatively higher weight values can be assigned to the active/positive compounds to promote their importance in the final feature weighting.

Our link-based framework can be written as follows. **a** represents the “active” system and **b** is the “inactive” system.
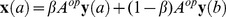
(10)

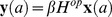
(11)

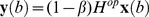
(12)



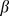
 is a class factor ranging from 0 to 1 (In the case that A^op^ or H^op^ involves 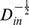 or 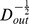, 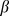 or (1-β) will be replaced by their square roots). It has impact on the accuracy and size of classifiers along with rules in the classifiers. Generally, in order to assign higher weight values to active/positive compounds, 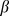 can be any value greater than 0.5. In our study, 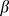 is set to 0.9.

Based on the comparison of implementations in [34], the following definitions of A^op^ and H^op^ are used.


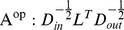
(13)


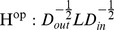
(14)

### 7. Associative Classification Mining

Let F = { f_1_, f_2_, …, f_n_} be a set of n distinct features and C be a list of classes { c_1_, c_2_,., c_m_}. D is a transaction/dataset over F and C. Each transaction/compound t_i_ contains a set of items f_1_, f_2_, …f_k_ 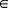
F and cj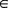 C. The set of items here is also called *itemset*. A classification association rule (CAR) is an implication of the form X 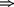 Y or X 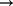
Y where X 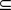 F and Y 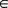 C. The *support* of the rule is the probability of transactions having both X and Y 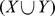 among all the presented cases. An itemset is *frequent* only if its support satisfies a minimum support θ. Additionally, the *confidence* of this rule is defined as the support of X and Y 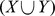
divided by the support of X which is the conditional probability Y is true under the circumstance of X. The process of discovering, pruning, ranking and selecting of CARs and applying them to classification is called *associative classification*.

### 8. Weighted Associative Classification Mining

For the weighted associative classification (WAC) [15]–[17], each feature f_i_ is associated with a weight w_i_ 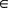 W = { w_1_, w_2_, …, w_n_}. A pair (f_i_, w_i_) is called a *weighted item*. Each transaction/compound is a set of weighted items plus the class type. The straightforward definition of *itemset weight* is:
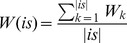
(15)W(*is*) is the weight of itemset and *is* is the itemset. The *weighted support* of itemset WS(*is*) is:
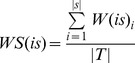
(16)T is total transactions and S is all the transactions containing the itemset. In the classical associative classification, the difference of significance of items is not taken into account. It is assumed that if the itemset is frequent, then all of its subsets should be frequent as well. This principle is called downward closure property (DCP). Given the compounds C1–C6, their features and the weight of the features (**Table 1** & **2**), if itemset {81, 83, 84} is frequent, then all its subsets {81}, {83}, {84}, {81, 83}, {81, 84} and {83, 84} must all be frequent. However, in WAC, provided the convenient definition (**equation 15** & **16**), the DCP does not hold. An itemset may be frequent even though some of its subsets are not frequent which can be illustrated in the following example (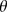 = 0.3). As shown in **Table 3**, the support of {83, 84} and {81, 83} are both 0.27 so they are not frequent.

Several frameworks are proposed to maintain the DCP property [15]–[22], [25]. Before introducing the framework, we define the *transaction weight* as:
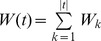
(17)
*t* is the transaction. We then define the **adjusted weighted support** as:
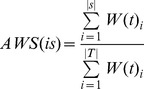
(18)The S and T are the same as above. This definition will ensure that if 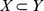 then 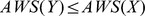 since any transaction containing Y will have X. By using the AWS, the DCP will not be violated. The discovered association rules are ranked, evaluated and pruned by using CBA approach [5]. The algorithm of PageRank based associative classification is given in **Figure 2** & **3**.

All the computations are carried out on a PC Q6600 2.4GHz with 6G memory running on the Windows 7 64bit operating system. The classifier is implemented in C#. To explore all possible rules, the mining is performed by using the following settings: MinSup (20%) and MinConf (70%) for AMES dataset; MinSup (1%) and MinConf (0%) for NCI-60 dataset. In all experiments, the maximum length of the rules is set to 4 and the maximum number of candidate frequent itemsets is 200,000. In the AMES data set, the SVM and RELIEF weighting method are applied for comparison. SVM and RELIEF are computed using Rapidminer 5.1 [42].

### 9. Model Assessment and Evaluation

The classification performance is assessed using 10-fold “Cross Validation” (CV) because this approach not only provides reliable assessment of classifiers but the result can be generalized well to new data. The accuracy of the classification can be determined by evaluation methods such as error-rate, recall-precision, any label and label-weight etc. The error-rate used here is computed by the ratio of number of successful cases over total case number in the test data set. This method has been widely adopted in CBA [5], CPAR [42] and CMAR [4] assessment.

## Results and Discussion

### 1. Comparison of Feature Weight and Rank

The comparison is performed on AMES dataset. For AMES dataset mining, the identification of features which are good for “positive” compounds are considered more preferable. So the “positive” here is treated as “active”. The weight generated by LAC is compared to that generated by frequency of the bits, SVM and RELIEF. **Figure 4** shows that results of RELIEF and SVM are very similar. To confirm this, a correlation analysis is performed by SPSS 19 [43]. **Table 4** shows at the 0.01 level (2-tailed), SVM and RELIEF, LAC and frequency are highly correlated as the coefficient is 0.949 and 0.958 respectively. The coefficients of SVM, RELIEF and LAC with frequency are greater than 0.75 indicating that all are correlated with frequency. Among them, LAC has the strongest correlation (0.947) with frequency. This is mainly caused by bit 3, 8, 11, 36 and 166. For bit 3, 8 and 11, since their frequencies are not 0, both LAC and frequency assign small weight values while for SVM and RELIEF the weight values are set to 0. On the contrary, the weight values of 36 and 166 are set to 0 for LAC and frequency but are not set to 0 in SVM and RELIEF. The correlation of LAC and frequency can be explained by the principle of link-based weighting–mutual reinforcement. As expected, the rank and weight of features in the LAC and frequency are different. In **Table 5**, all features are ordered by ascending weight. 69 features (bold) are promoted and 61 features (*) are demoted while the rest remains unchanged in LAC. Generally, higher frequency will lead to higher “authority” resulting bigger weight (**Figure 4**). For example, bit 135 has high weight in both frequency and LAC; bit 127 and 141 are much bigger in LAC (red data label) than in frequency (black data label) since most of their connections are “active” compounds (58.6% and 56.6% respectively). **Table 5** is the rank of the features in each scheme respectively. The bigger the number, the higher the rank is and the more important the feature is. Some features (bold) have a relatively lower rank in frequency; they may get higher ranks due to the promotion from connecting to compounds having higher “rank” values. Likewise, features (*) connected to many “bad” compounds may be degraded. The promotion or demotion depends on the number and type of its connections.

### 2. Comparison of Accuracy of Classification

The average accuracies of frequency, LAC, RELIEF, SVM and CBA are 90.11%, 91.57%, 89.05%, 89.26% and 90.63% respectively (**Table 6**). The major purpose of WACM is to find more rules containing interesting items, in other word, items with higher significance, while trying to achieve high accuracy at the same time. Most of current comparisons of performance between WARM and traditional ARM are focused on time and space scalability, such as number of frequent items, number of interesting rules, execution time and memory usage [18]–[20], [43]–[45]. The results showed that the difference between WARM and ARM are minor. The comparison of WACM and traditional ACM is scant due to the lack of easily accessible weighted association classifiers. Soni et al [46] compared their WACM results with those generated by traditional ACM methods–CBA [5], CMAR [4] and CPAR [47] on three biomedical datasets, and their results showed that WACM offered the highest average accuracy. In our study, among all four weighted schemes and CBA, LAC has the highest accuracy.

### 3. Comparison of Classifiers

There are 10 models generated for each weighting scheme and we are interested in the comparison between the classifiers of CBA and LAC. Model 1 is used as an example and there are 30 rules in the classifier of frequency and 132 in that of LAC. Among them, 14 rules are exclusively in the frequency classifier, 116 only in LAC classifier and 16 rules are shared by both. **Table 7** shows that among the top 20 rules, 11 rules are shared by both classifiers, 9 rules (*) are only in the classifier of frequency and none of the top 20 rules (bold) are included in the classifier of frequency. All rules are ordered based on the CBA definition. During the classification, the match of the new compounds starts from the first and will stop immediately as long as there is a hit. As a result, although those 11 rules are in both classifiers, they may have different impacts on the final result of classification.

### 4. Rule Interpretation

Our recently submitted paper [48] showed that the rules generated by associative classification based on chemical fingerprints and properties can be interpreted by chemical knowledge and shed a light on the molecule design. In this study, we focus on the analysis of association rules generated by LAC using the bio fingerprint (NCI-60 dataset). The analysis for those generated by frequency can be done in the same manner. The accuracy of both frequency and LAC are 99.93% (**Table 6**) and the average size of the classifier is around 350 rules.

For all ten models, the top 5 rules are the same but with different order, support and confidence. The intuitive explanation of Rule 1 in **Table 8** is that if compound is inactive to MCF7 and HL60 (TB) then it will be inactive to T47D at the same time. The adjusted weighted support of this rule is 29.1% and weighted confidence is 95.9%. Among the 5,937 compounds, 1730 compounds are covered by this rule. All these cell lines in the top 5 rules fall into two categories: a) breast cancer and b) Leukemia. On one hand, it means that there are many compounds which are inactive neither to breast cancer cell lines nor to Leukemia cell lines; on the other hand, it suggests that there might be some associations between these two types of cancers. [49], [50] clustered the cell lines based on their gene expression data, their results also indicated that the cell lines in these two categories were clustered into one or their clusters were very close to each other. The association of MCF7 and T47D is not surprising as they belong to the same category–breast cancer. The rules here may also provide a potential direction of the drug resistance of breast cancer and leukemia. [50]–[52] discovered a novel ABC transporter, breast cancer resistance protein (BCRP). This transporter was termed breast cancer resistance protein (BCRP) because of its identification in MCF-7 human breast carcinoma cells. The drug-sensitive cells become drug-resistant cells after transfection or overexpression of BCRP. They also found that relatively high expression of BCRP mRNA were observed in around 30% acute myeloid leukemia (AML) cases and suggested a novel mechanism of drug resistance in leukemia.

A hybrid feature set integrating the chemical fingerprint and bio fingerprint is generated by combining the MDL public keys and the bio fingerprint. Since we are only interested in the compounds which are active against tumor cell lines, the “inactive” value of the bioassay is treated as a feature of “not existed” in the compound. This also helps to treat the chemical fingerprint and the bio fingerprint equally.

The average accuracy of the classification is 99.7% (**Table 6**). For rules in the final classifier, for example, (A, B → Active), it will be converted to (A associate Active) and (B associate Active). All the rules are transferred and plotted by Cytoscape 2.8.2 [53]. To make it clearer, nodes with degree less than 10 are removed. **Figure 5** shows that generally compounds actively against MDA-MB-231/ATCC, TK-10, OVCAR-4, UACC-257, HOP-92, EKVX, NCI-H226 will also active to T-47D. Chemical features: bit 46(Br), 51 (CSO), 58 (QSQ), 65 (CN), 127 and 111 (NACH2A) are related to active or inactive depending on what other features it coexists with. There are other features which mainly related to inactive.

The top 2 rules in the classifier indicate that compounds containing phosphorus and active to MCF7 or SK-MEL-2 will be active to T-47D too (**Table 9**). 22 out of 23 compounds match both rule 1 and 2. Among them, the once abandoned drug NSC 280594 (triciribine) attracts much attention and undergoes phase I trial due to its potential possibility of against a common cancer-causing protein [53]–[55]. These rules reveal that phosphorus might be an important chemical structure for anti-cancer drugs.

### Conclusions

In this paper, we describe a novel link-based feature weighting framework for datasets without pre-assigned weight information. This algorithm employs a unified framework which integrates the advantage of HITS and PageRank–the mutual reinforcement and normalized weights–to derive useful weights. It utilizes connectivity and connection type information. Combined with a weighted support scheme, it offers an effective way to find the useful associations by taking into account both the significance of occurrence and the quality of features. The latter is included by connections to the transactions.

Based on this new weight scheme, a CBA based classifier, LAC, is developed. The classifier is applied to two cases: the chemical fingerprint featured dataset and the bio-fingerprint featured dataset. Our experimental results show that although the weighting differs from the traditional RELIEF and SVM, it is able to capture the important features and afford good results. Especially for some sparse dataset, some significant features can be discovered by this link-based analysis which will be ignored by other methods.

The link-based classifier discovers interesting associations of bioactivities with chemical features and potential relationships among diseases, for instance, relationship between phosphorus and bioactivity against T47D and potential relationship between breast cancer and leukemia. Our next step will apply this method to large semantic data sets to mine information from the RDF resources such as ChEMBL [56] and KEGG [57].
